# Diode Laser-Assisted Surgical Therapy for Early Treatment of Oral Mucocele in a Newborn Patient: Case Report and Procedures Checklist

**DOI:** 10.1155/2018/3048429

**Published:** 2018-04-24

**Authors:** Marina Consuelo Vitale, Maria Francesca Sfondrini, Giorgio Alberto Croci, Marco Paulli, Lorenzo Carbone, Paola Gandini, Andrea Scribante

**Affiliations:** ^1^Unit of Orthodontics and Paediatric Dentistry, Section of Dentistry, Department of Clinical, Surgical, Diagnostic and Paediatric Sciences, University of Pavia, Pavia, Italy; ^2^Unit of Anatomic Pathology, Department of Molecular Medicine, University of Pavia and Fondazione IRCCS Policlinico San Matteo, Pavia, Italy

## Abstract

Mucocele (also known as ranula or salivary gland mucous cyst) of the newborn is a lesion present on the intraoral cavity, with the potential to interfere with respiration and feeding. In the present report, a case of mucocele in a 4-month female patient has been described. As conventional surgery can be followed by several complications such as intraoperative bleeding, difficulties in wound healing, and maintenance of sterility during surgery, in the present case, the use of diode laser has been planned. A topic anesthesia with lidocaine gel was performed. A diode laser (810 nm wavelength, continuous wave mode, power output of 3 watt, and 0.4 mm diameter fiber optic) was set for excising the lesion. The tip was directed at an angle of 10 to 15°, moving around the base of the lesion with a circular motion. The procedure was completed in 3 minutes. The patient was visited with a follow-up of 2 weeks and 4 months after excision. The intraoral wound healed without complications, and no signs of infection or mass recurrence were noted. The histopathological examination confirmed the diagnosis of mucocele. On the basis of the results of the present case report, the use of diode laser can be easily performed also in a noncompliant newborn patient for successful excision of mucocele lesions, and checklist of clinical procedures has been described.

## 1. Introduction

Mucocele (also known as ranula or salivary gland mucous cyst) of the newborn has been extensively reported in Literature [1–3]. This benign lesion of the oral cavity could potentially interfere with respiration and feeding [4]. This condition in neonates represents a situation often creating anxiety and apprehension among parents. Early examination and prompt diagnosis can aid in prudent management and serve as baseline against the future course of the disease [5]. It is difficult for the clinician to establish an accurate diagnosis based only on clinical symptoms, so a biopsy with histological examination is necessary to exclude other lesions [6].

Conventional treatment of mucocele involves a surgical approach for excision with general or local anesthesia. This procedure can be followed by several complications such as intraoperative bleeding, difficulties in wound healing, and maintenance of sterility during surgery [7].

Some authors described the efficacy of lasers in the treatment of oral tissues problems for photodynamic therapy [8, 9] and surgical procedures [1, 10, 11]. Diode laser (with wavelengths varying between 800 and 980 nm) is poorly absorbed by hard dental tissue, is safe, and well indicated for soft oral tissue surgeries for cutting, vaporization, curettage, blood coagulation, and hemostasis in the oral region [12].

To our knowledge, case reports presented in Literature concerning chairside laser treatments of mucocele are related to child, adolescent, and adult patients [1], but no case reports have been presented with newborn patients yet. The approach in newborn patients, due to their lack of compliance, has been reported in general anesthesia, with the relative problems and risks [4].

Therefore, the aim of the present manuscript was to present a case report about the use of a diode laser for chairside treatment of a mucocele in a newborn patient as a conservative and nonstressful method. The case has been described, and a checklist of clinical operative procedures has been proposed.

## 2. Case Report

### 2.1. Diagnosis and Etiology

**Figure 1:**
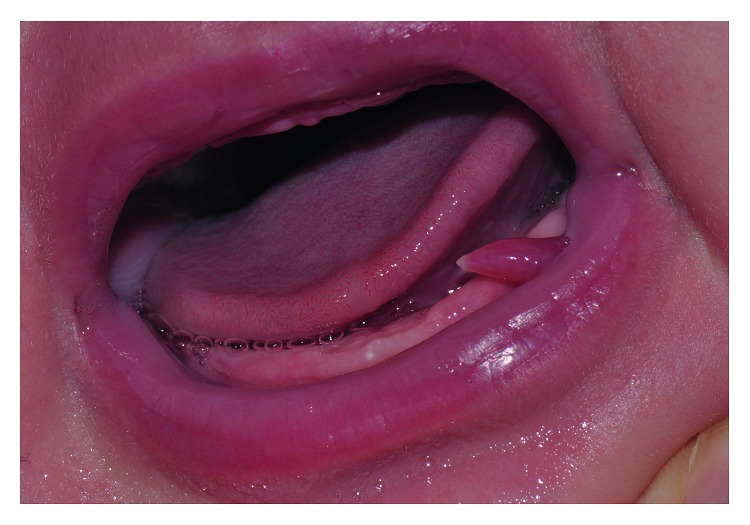
Initial intraoral photograph (age: 4 months).

A baby female, aged 4 months, was referred to our Orthodontics and Paediatric Dentistry Unit. The patient was receiving breast-feeding since birth. Her parents reported, during the first months of life, the spontaneous formation of a pink oblong vesicle in the left internal part of the lower lip (Figure 1). The color [13], the localization [14], and the shape [15] have been considered coherent with a mucocele lesion; however, a conclusive diagnosis requires histopathological examination. As spontaneous regression of these oral lesions has been reported [16], the parents planned only a further control.

**Figure 2:**
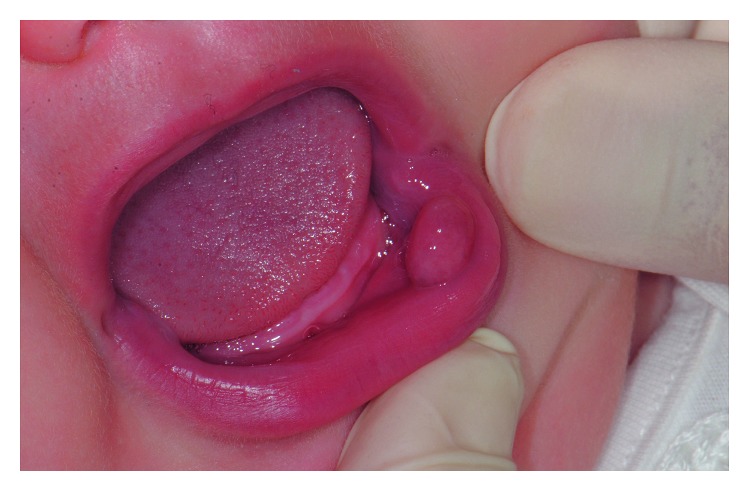
Intraoral photograph (age: 6 months).

After a month, the patient returned, and the lesion had a significant growth with a shape modification and appeared as a more regular bulla (Figure 2). Therefore, the excision intervention was planned. The size of lesion in the moment of surgery was about 10 mm × 6 mm.

### 2.2. Treatment Objectives

The objective of the treatment was mucocele treatment with topic anesthesia and laser-assisted excision.

### 2.3. Treatment Alternatives

The first possible alternative to the treatment was the delay of the intervention, thus planning only further controls, with the risk of lesion growth and feeding problems. Another alternative consisted in a conventional lancet surgical approach, with the consequent risks of intra- and postoperative bleeding and potential difficulties in wound healing. The last option was marsupialization that would allow lesion drainage without excision, but this technique is more fitted for larger lesions.

### 2.4. Treatment Progress

**Figure 3:**
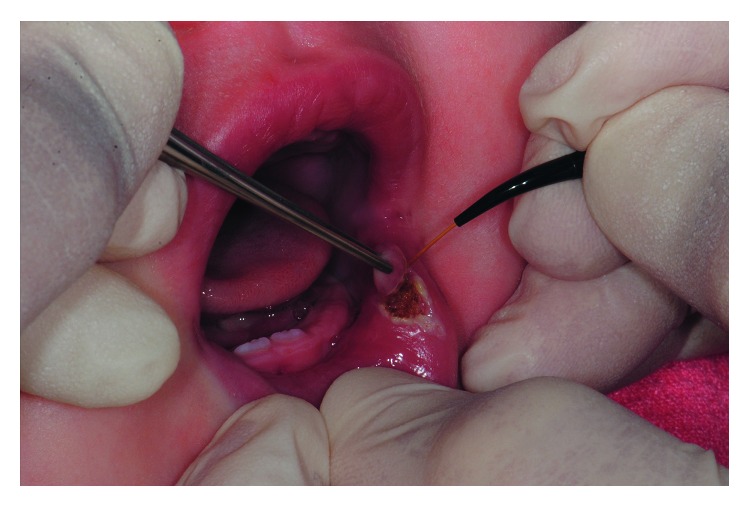
Laser-assisted lesion excision (age: 6 months).

**Figure 4:**
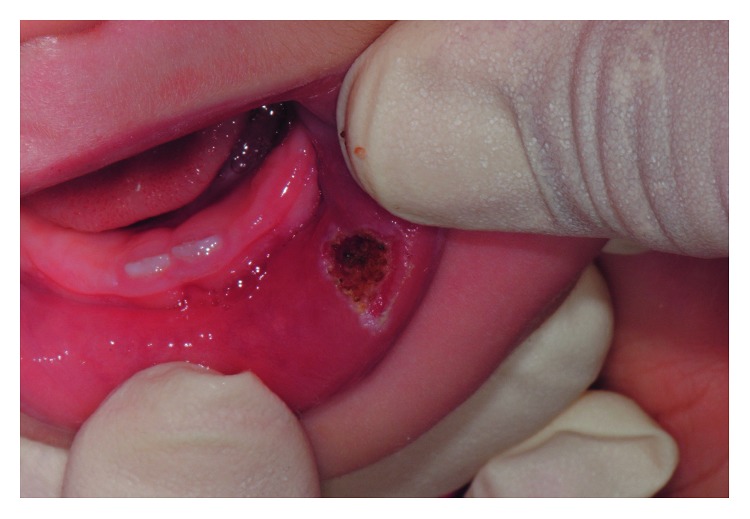
Intraoral photograph immediately after intervention.

Written informed consent was obtained from the patient's parents to proceed with lesion excision with laser surgery. The patient and the whole staff wore protective glasses to prevent eye damage [17]. Local topic anesthesia was performed with lidocaine gel local application for one minute. Diode laser (Diode Laser, DMT Lissone, Italy) at 810 nm wavelength, continuous wave mode with a power output of 3 watt, and a 0.4 mm diameter fiber optic were set for excising the lesion. The tip was directed at an angle of 10 to 15°, moving around the base of the lesion with a circular motion [18] (Figure 3). It took 3 minutes to complete the procedure. The diode laser provided a combination of clean cutting of the tissue and hemostasis (Figure 4). The patient was discharged with necessary postoperative instructions for maintenance of good oral hygiene and keeping the area clean. No additional analgesic or antibiotic was recommended.

After excision, the lesion was immersed in formalin and then was sent to histopathologic service for evaluation.

### 2.5. Treatment Results

**Figure 5:**
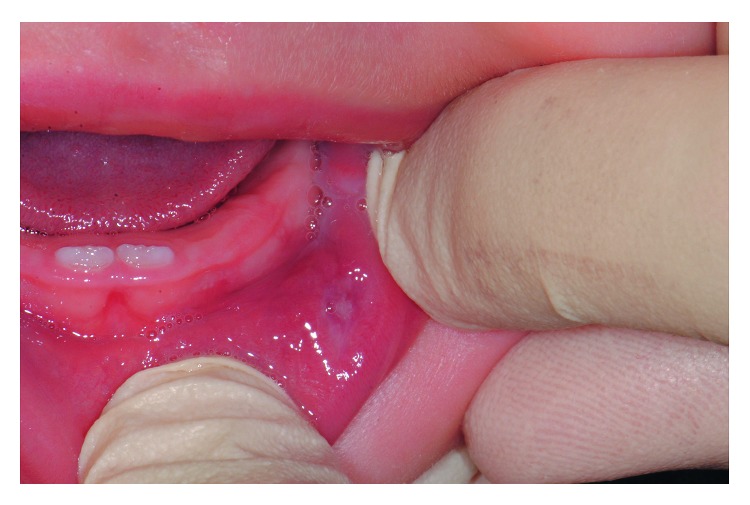
Healing (age: 6.5 months).

**Figure 6:**
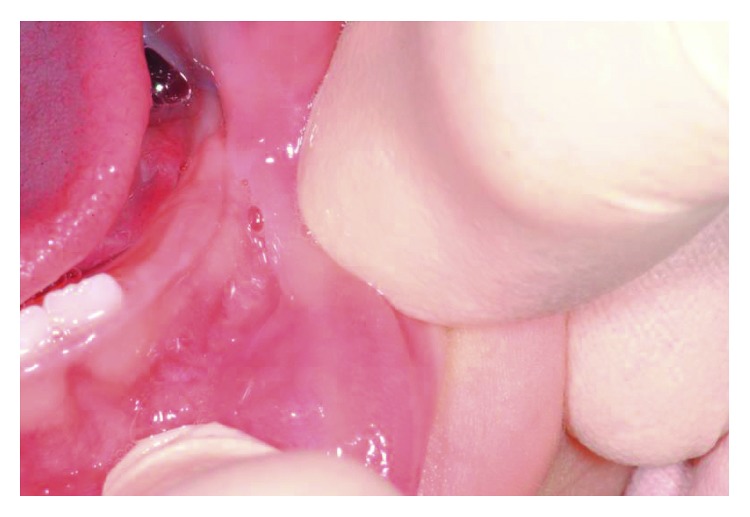
Follow-up (age: 10 months).

After excision, the patient had no signs of respiratory distress and no feeding difficulty was reported from the parents. The patient was visited with a follow-up of 2 weeks and 3 months. After 2 weeks follow-up (Figure 5), the intraoral wound healed without complications and no signs of infection or mass recurrence were noted. After 4 months (Figure 6) follow-up, the lesion healed completely and the patient had a functionally and developmentally normal mucosa without lesion recurrence. The patient demonstrated age-appropriate weight gain.

**Figure 7:**
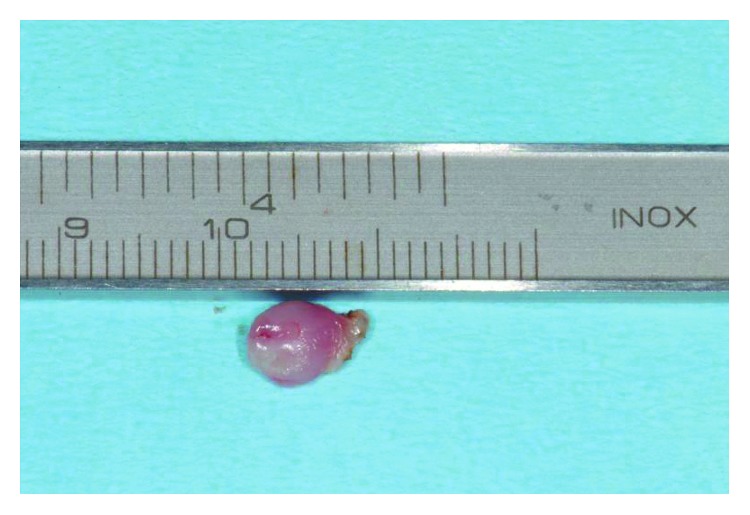
The lesion measured about 1 cm maximal length, displayed a polypoid fashion, and was covered by a smooth mucosal layer.

**Figure 8:**
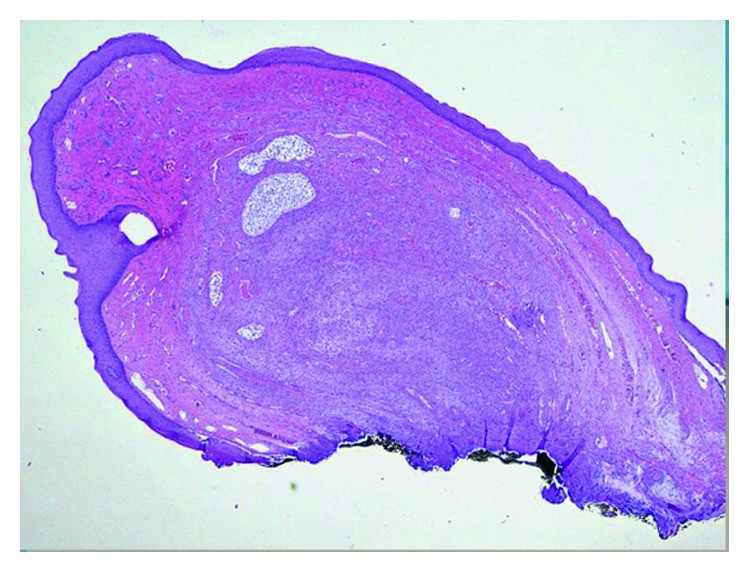
Histologic examination revealed a process deep seated within the submucosal connective tissue (HE, ×2 magnification).

**Figure 9:**
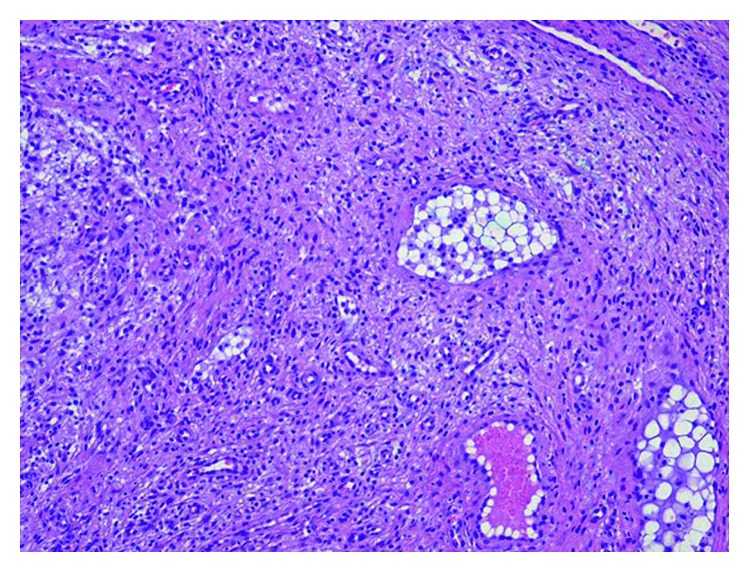
The lesion consisted of newly formed capillary vessels intermingled with a chronic, lymphohistiocytic inflammatory infiltrate and associated with deposition of extracellular mucin (HE, ×10 magnification).

**Figure 10:**
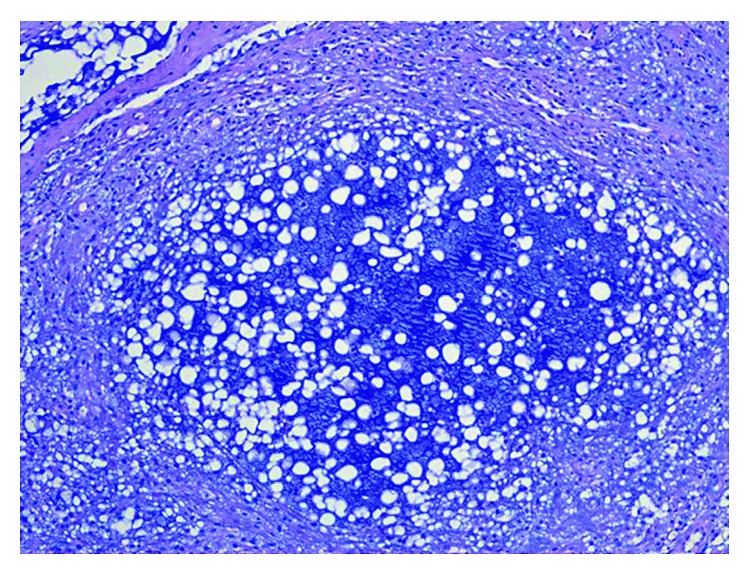
Extracellular mucin resulted Alcian blue positive (Alcian-PAS, ×20 magnification).

The histopathological examination confirmed the initial clinical diagnosis of mucocele. Grossly, the lesion displayed a polypoid fashion and was covered by a smooth mucosal layer (Figure 7). Histologic examination revealed a process deep seated within the submucosal connective tissue (Figure 8), consisting of newly formed capillary vessels intermingled with a chronic, lymphohistiocytic inflammatory infiltrate and associated with deposition of extracellular mucin (Figure 9), the latter which resulted Alcian blue positive (Figure 10). Thus, the histopathologic picture was consistent with stromal reaction to extravasated mucin, possibly related to an injured, salivary gland mucous cyst (mucocele).

## 3. Discussion

In the present report, a mucocele lesion in a baby female, aged 6 months, was treated with laser excision. Clinically, this lesion usually appears as an asymptomatic vesicle or bulla with a pink or bluish color [15]. The size can extend from 1 mm to some centimeters [19] and are most frequently located on the lower lip [14]. Mucocele lesions can be of two types: extravasation and retention mucoceles, the former affecting the lower lip most frequently [3].

In the present case, the lesion was pink, bulla-shaped with a size of 10 mm approximately, and was situated on the lower lip. These characteristics are coherent with typical aspects of these lesions, and for this reason, the diagnosis before the histologic report was of “suspected mucocele.” This lesion is commonly due to alterations in the minor salivary glands and is occurring with a prevalence of 0.2 cases per 1,000 persons. Approximately 2.7% of patients are under the age of one [20]. However, the real frequency of oral mucocele is difficult to calculate, as many of these lesions recede spontaneously [16].

As occasional spontaneous regression has been reported for these lesions [21], the frequent monitoring for regression may be used as a management option. However, if the lesion continues growing or if it interferes with respiration or deglutition, conservative excision under anesthesia is recommended as the traditional treatment [13]. For these reasons, in the present report, parents, after the first visit, decided to plan only a control after a couple of months. During the second control, the lesion showed a significant increase in dimensions and volume so the parents accepted to plan an intervention.

We decided to avoid both marsupialization that is often planned for larger lesions [15] and conventional lancet surgical approach, with the consequent risk of bleeding and potential difficulty in wound healing [1]. Therefore, a laser-assisted excision was planned. The advantages of this technique over conventional surgical approach are multiple.

The first advantage is related to anesthesia. From a clinical point of view, a topic on anesthesia has been reported to be sufficient for pain control during laser application on soft tissue as lidocaine-containing products play an integral role in mucous anesthesia by providing patient comfort with minimal side effects [22]. This possibility allows the clinicians to avoid more invasive techniques, such as the injection, the sedation, and the general anesthesia.

The second advantage is related to procedure speed. The laser-assisted procedure is reported to require significant less time than the conventional approach during surgical excision of oral lesions, thus reducing patient discomfort [10]. Indeed, pain and time reduction are crucial aspects during therapy, especially for paediatric patients.

The third advantage is the lack of bleeding. Lasers provide cut and coagulation in one time so no bleeding is present during intervention. Moreover, no sutures are required at the end of the surgical procedure [23]. Previous authors showed laser-assisted mucocele excision [1, 10, 11, 24–27]. A brief review of the dosimetry and techniques used in previous clinical studies is reported in Table 1.

In the present study, after excision, the parents reported no problems in feeding, the intraoral alveolar wound healed without complications, and no signs of infection or mass recurrence were noted. Laser-assisted excision of oral lesions has been extensively reported in Literature, and no particular side effects have been showed [3]. A risk of recurrence is reported with mucoceles [1]. In the present report, at a follow-up of 4 months, the lesion showed no recurrence. Moreover, in the present report, the minor salivary gland that caused the mucocele has been presumably removed. However, the cauterization produced by laser either in the tissue removed and in the oral mucosa of the patient does not allow the total excision certainty. Therefore, a recurrence risk is always present, with a 5% to 7% risk [1, 15] for the anatomic region that has been treated.

On the basis of the present case report and Literature review, in clinical cases with a mucocele suspect, a procedure checklist could be considered as follows:

- Check the lesion coherence with mucocele characteristics (asymptomatic, bulla shape, pink or bluish color, 1 mm to several cm diameter, and location on the lower lip predominantly) [15].
- If the lesion does not heal spontaneously after two weeks, the excision could be planned [21].
- Intervention consist in topic anesthesia [22], protective glasses wearing [17], and lesion excision [18] with either diode (continuous or pulsed mode, 810 nm to 940 nm wavelength, and 1.3 to 3 W power output), or CO_2_ (continuous mode, 10.6 *µ*m wavelength, and 3 W to 5 W power output) laser with a 0.4 mm fiber diameter and tip angulation of 10°–15°approximately.
- Surgical piece can be stored in formalin, if anatomopathological exam is needed.
- Check for recurrence after 1 to 6 months.

Certainly, before excision planning, the evaluation of lesion dimension and location is crucial. In fact, bigger manifestations (with a diameter of some centimeters) and lesions localized in hard-to-reach areas (such as the sublingual mucosa) are deserved for a conventional general surgery approach.

## 4. Conclusions

After topical anesthesia, diode laser excision of mucocele lesions could be applied also in newborn patients with a few-minute surgical approach. This procedure is particularly safe and effective.

## Figures and Tables

**Table 1:** Brief review of the dosimetry and techniques used in previous clinical reports.

Authors	Laser	Mode	Power setting	Wavelength
Kato and Wijeyeweera [10]	CO_2_	Continuous	3 W or 4 W	10.6 *µ*m
Pedron et al. [24]	Diode	Continuous	2 W	810 nm
Wu et al. [1]	CO_2_	Continuous	5 W	NA
Agarwal et al. [25]	Diode	Pulsed (10 ms)	1.3 W	940 nm
Chinta et al. [26]	Diode	Pulsed (50 ms)	2 W	810 nm
Ramkumar et al. [11]	Diode	Continuous	1.5 W	940 nm
Ahad et al. [27]	Diode	Pulsed (30 ms)	2 W	810 nm

## References

[1] Wu C. W., Kao Y. H., Chen C. M., Hsu H. J., Chen C. M., Huang I. Y. (2011). Mucoceles of the oral cavity in pediatric patients. *Kaohsiung Journal of Medical Sciences*.

[2] Martins-Filho P. R., de Santana Santos T., Piva M. R. (2015). A multicenter retrospective cohort study on pediatric oral lesions. *Journal of Dentistry for Children*.

[3] Patil S., Rao R. S., Majumdar B., Jafer M., Maralingannavar M., Sukumaran A. (2016). Oral lesions in neonates. *International Journal of Clinical Pediatric Dentistry*.

[4] George M. M., Mirza O., Solanki K., Goswamy J., Rothera M. P. (2015). Serious neonatal airway obstruction with massive congenital sublingual ranula and contralateral occurrence. *Annals of Medicine and Surgery*.

[5] Rodríguez H., De Hoyos Parra R., Cuestas G., Cambi J., Passali D. (2014). Congenital mucocele of the tongue: a case report and review of the literature. *Turkish Journal of Pediatrics*.

[6] Adegun O. K., Tomlins P. H., Hagi-Pavli E., Bader D. L., Fortune F. (2013). Quantitative optical coherence tomography of fluid-filled oral mucosal lesions. *Lasers in Medical Science*.

[7] Fritz G. R., Stern P. J., Dickey M. (1997). Complications following mucous cyst excision. *Journal of Hand Surgery*.

[8] Tsai J. C., Chiang C. P., Chen H. M. (2004). Photodynamic therapy of oral dysplasia with topical 5-aminolevulinic acid and light-emitting diode array. *Lasers in Surgery and Medicine*.

[9] Barcessat A. R., Huang I., Rosin F. P., dos Santos Pinto D., Maria Zezell D., Corrêa L. (2013). Effect of topical 5-ALA mediated photodynamic therapy on proliferation index of keratinocytes in 4-NQO-induced potentially malignant oral lesions. *Journal of Photochemistry and Photobiology B: Biology*.

[10] Kato J., Wijeyeweera R. L. (2007). The effect of CO_2_ laser irradiation on oral soft tissue problems in children in Sri Lanka. *Photomedicine and Laser Surgery*.

[11] Ramkumar S., Ramkumar L., Malathi N., Suganya R. (2016). Excision of mucocele using diode laser in lower lip. *Case Reports in Dentistry*.

[12] Amaral M. B., Freitas I. Z., Pretel H., Abreu M. H., Mesquita R. A. (2012). Low level laser effect after micro-marsupialization technique in treating ranulas and mucoceles: a case series report. *Lasers in Medical Science*.

[13] Conrad R., Perez M. C. (2014). Congenital granular cell epulis. *Archives of Pathology & Laboratory Medicine*.

[14] Shapira M., Akrish S. (2014). Mucoceles of the oral cavity in neonates and infants–report of a case and literature review. *Pediatric Dermatology*.

[15] Piazzetta C. M., Torres-Pereira C., Amenábar J. M. (2012). Micro-marsupialization as an alternative treatment for mucocele in pediatric dentistry. *International Journal of Paediatric Dentistry*.

[16] Oliveira D. T., Consolaro A., Freitas F. J. (1993). Histopathological spectrum of 112 cases of mucocele. *Brazilian Dental Journal*.

[17] Sweeney C. (2008). Laser safety in dentistry. *General Dentistry*.

[18] Asnaashari M., Azari-Marhabi S., Alirezaei S., Asnaashari N. (2013). Clinical application of 810 nm diode laser to remove gingival hyperplasic lesion. *Journal of Lasers in Medical Sciences*.

[19] Mínguez-Martinez I., Bonet-Coloma C., Ata-Ali-Mahmud J., Carrillo-García C., Peñarrocha-Diago M., Peñarrocha-Diago M. (2010). Clinical characteristics, treatment, and evolution of 89 mucoceles in children. *Journal of Oral and Maxillofacial Surgery*.

[20] Gatti A. F., Moreti M. M., Cardoso S. V., Loyola L. V. (2001). Mucus extravasation phenomenon in newborn babies: report of two cases. *International Journal of Paediatric Dentistry*.

[21] Ritwik P., Brannon R. B., Musselman R. J. (2010). Spontaneous regression of congenital epulis: a case report and review of the literature. *Journal of Medical Case Reports*.

[22] Greveling K., Prens E. P., Liu L., van Doorn M. B. (2017). Non-invasive anaesthetic methods for dermatological laser procedures: a systematic review. *Journal of the European Academy of Dermatology and Venereology*.

[23] Karimi A., Sobouti F., Torabi S. (2016). Comparison of carbon dioxide laser with surgical blade for removal of epulis fissuratum. A randomized clinical trial. *Journal of Lasers in Medical Sciences*.

[24] Pedron I. G., Galletta V. C., Azevedo L. H., Corrêa L. (2010). Treatment of mucocele of the lower lip with diode laser in pediatric patients: presentation of 2 clinical cases. *Pediatric Dentistry*.

[25] Agarwal G., Mehra A., Agarwal A. (2013). Laser vaporization of extravasation type of mucocele of the lower lip with 940-nm diode laser. *Indian Journal of Dental Research*.

[26] Chinta M., Saisankar A. J., Birra C., Kanumuri P. K. (2016). Successful management of recurrent mucocele by diode laser and thermoplasticised splint as an adjunctive therapy. *BMJ Case reports*.

[27] Ahad A., Tandon S., Lamba A. K., Faraz F., Anand P., Aleem A. (2017). Diode laser assisted excision and low level laser therapy in the management of mucus extravasation cysts: a case series. *Journal of Lasers in Medical Sciences*.

